# Intake of Dietary Fiber From Grains and the Risk of Hypertension in Late Midlife Women: Results From the SWAN Study

**DOI:** 10.3389/fnut.2021.730205

**Published:** 2021-09-16

**Authors:** Peng Du, Kaifeng Luo, Yali Wang, Qi Xiao, Jiansheng Xiao, Yong Li, Xingjian Zhang

**Affiliations:** ^1^Department of General Surgery, The First Affiliated Hospital of Nanchang University, Nanchang, China; ^2^Department of Pathology, The Affiliated Stomatological Hospital of Nanchang University, Nanchang, China

**Keywords:** dietary fiber, blood pressure, hypertension, midlife women, smooth curve

## Abstract

**Background:** The possible effects of dietary fiber intake on hypertension have not been clarified fully. The association of dietary fiber intake with hypertension risk in midlife women was analyzed in this study.

**Methods:** Baseline data were obtained from the Study of Women's Health Across the Nation (SWAN). Smooth curve, linear regression, and logistic regression analyses were performed to investigate the associations of four indices of daily dietary estimate (DDE) of dietary fiber (dietary fiber intake, dietary fiber intake from beans, dietary fiber intake from vegetables/fruit, and dietary fiber intake from grains) with blood pressure in midlife women. For this research purpose, diastolic blood pressure (DBP) ≥90 mmHg was defined as diastolic hypertension, and systolic blood pressure **(**SBP) ≥140 mmHg was defined as systolic hypertension.

**Results:** This study included 2,519 participants with an average age of 46. The smooth curve showed approximate negative correlations between three fiber indices (DDE dietary fiber, DDE fiber from vegetables/fruit, and DDE fiber from grains) and blood pressure, including DBP and SBP (all *P* < 0.005). There were also approximate negative correlations between two fiber indices (DDE dietary fiber and DDE fiber from grains) and the risk of diastolic hypertension and systolic hypertension (all *P* < 0.05). Furthermore, multiple linear regression analysis suggested that DDE dietary fiber (Sβ = −0.057, 95% CI −0.194 – −0.012, *P* = 0.027), DDE fiber from vegetables/fruit (Sβ = −0.046, 95% CI −0.263 – −0.007, *P* = 0.039), and DDE fiber from grains (Sβ = −0.073, 95% CI −0.600 – −0.099, *P* = 0.006, Model 4) were still negatively correlated with DBP after adjusting for confounding factors. Only DDE fiber from grains was independently and negatively associated with SBP (Sβ = −0.060, 95% CI −0.846 – −0.093, *P* = 0.015) after these same confounding factors were adjusted for. Importantly, multiple logistic regression analysis suggested that only higher DDE fiber from grains was independently associated with a reduced risk of diastolic hypertension (OR = 0.848, 95% CI 0.770–0.934, *P* = 0.001, Model 4) and systolic hypertension (OR = 0.906, 95% CI 0.826–0.993, *P* = 0.034, Model 4) after the adjustments were made for confounding factors.

**Conclusions:** We found that dietary fiber intake, especially DDE fiber from grains, contributes to a lower risk of systolic hypertension and diastolic hypertension in midlife women.

## Introduction

Patients with hypertension tend to experience headaches, dizziness, and other symptoms for decades, and the damage caused by hypertension to the cardiovascular system is continuous and aggravating (1, 2). High blood pressure that is not well-controlled contributes significantly to an increased risk of serious diseases, such as myocardial infarction, ischemic and hemorrhagic stroke, aortic dissection or aneurysm, chronic kidney disease, and peripheral artery disease (3). There are many risk factors that are known to be associated with hypertension, such as age, heredity, environment, and lifestyle (4). Unhealthy lifestyles, including a high-salt diet, lack of exercise, fatigue, and high pressure from work and other areas of life, are the most important modifiable factors for developing hypertension (5). Using antihypertensive drugs can alleviate damage to target organs by hypertension and prevent adverse cardiovascular events. However, many individuals are either not aware of their blood pressure condition or are not aware of their true blood pressure values due to inadequate or discontinued antihypertensive treatment. Furthermore, although there are vast options for antihypertensive drugs, some patients with hypertension have difficulty controlling their blood pressure within the normal range by using current drugs, motivating further research in this field. Thus, early prevention of the formation of hypertension is still particularly important at present.

Importantly, the positive effects of dietary fiber supplementation on cardiovascular protection have been recognized for a few years (6–11). Some investigations on the role of dietary fiber intake in patients with hypertension have also been reported. For instance, one previous study reported an association of fiber intake with the prevention of cardiovascular disease, including hypertension (6). Oat, a fiber-rich food, has also been found to have beneficial effects on controlling blood pressure among patients with hypertension (7). However, studies on the exact relationship between dietary fiber intake and hypertension risk are few, and it has been studied inadequately (6). Lifestyle and eating habits in the middle-aged population tend to cause people to be at high risk of hypertension.

Accordingly, in this study, data analysis from a subset of the Study of Women's Health Across the Nation (SWAN) was performed to evaluate the relationships of four indices of daily dietary estimate (DDE) of dietary fiber (dietary fiber intake, dietary fiber intake from beans, dietary fiber intake from vegetables/fruit, and dietary fiber intake from grains) with blood pressure in midlife women.

## Methods

### Study Design and Participants

**Figure 1:**
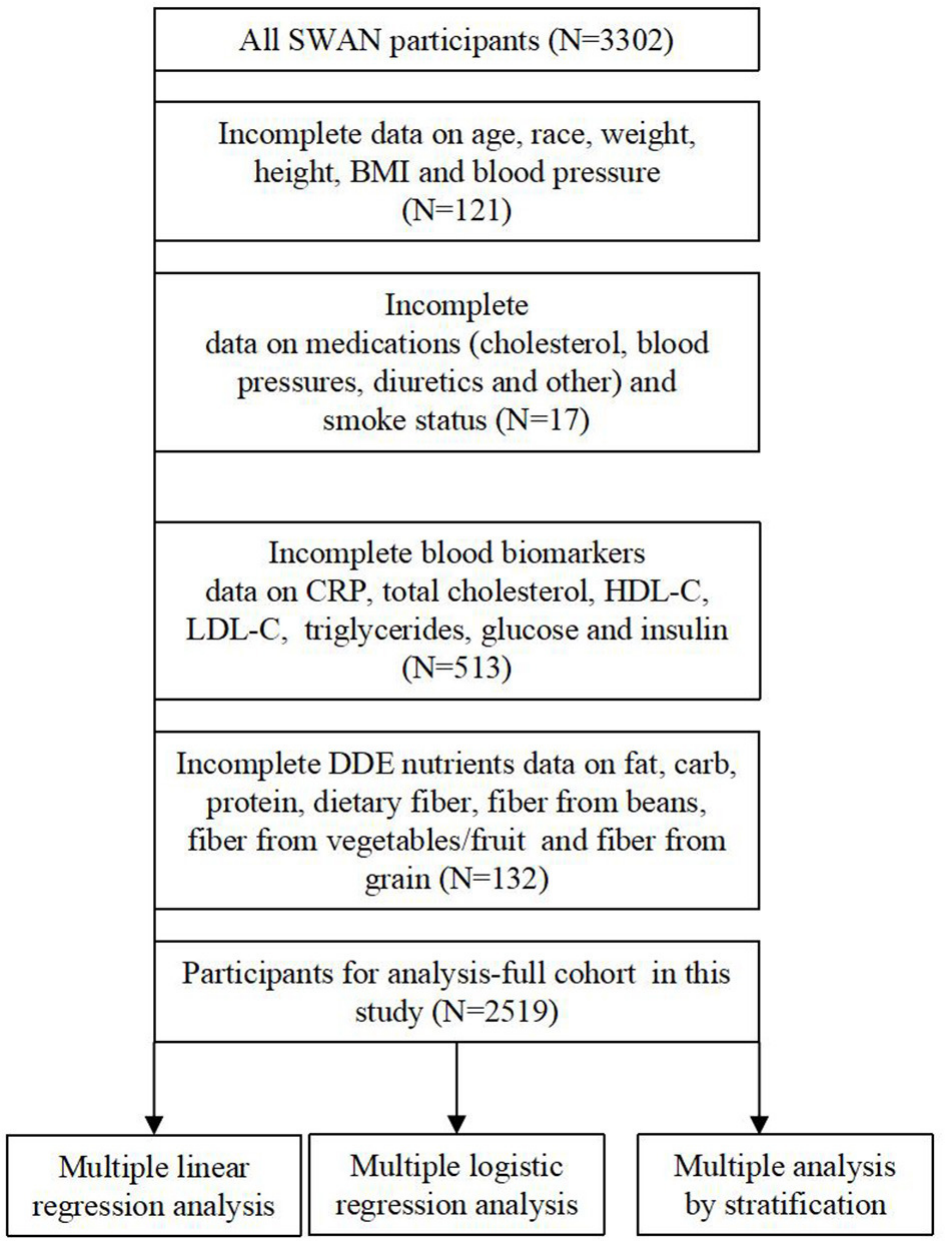
Flow chart of the included participants.

To analyze the baseline data from the SWAN study, a longitudinal, multicenter, and population-based study of the natural history of late midlife women was performed. Between 1995 and 1997, a telephone screening interview was used to determine the eligibility of an individual for the study cohort. Participants from community-based samples were collected at seven collection points across the United States using various sampling frames and recruitment strategies. In summary, 16,065 women completed the telephone screening interview, and only 3,302 (20.6%) women were recruited to the SWAN cohort at baseline. Women who met the inclusion criteria were as follows: (1) had a range of ages from 42 to 53 years; (2) had not used reproductive hormones in the previous 3 months; and (3) had an intact uterus and at least one intact ovary. The institutional review boards at all the sites approved the study protocol, and all included individuals at each site gave informed consent. The SWAN study contained detailed information regarding demographic characteristics, lifestyle, self-reported health, health examination, and medical history. For research purposes, 783 women were excluded due to missing information. A total of 2,519 women were included in this study. The exclusion criteria are described in detail in Figure 1.

### Measurement and Calculation of Blood Pressure

The measurement of blood pressure in all the included subjects was carried out according to a standardized protocol (12). Subjects did not consume caffeinated beverages or smoke for at least 30 min before measuring blood pressure. The measurements were performed with readings taken on the right arm, with the participant seated and feet flat on the floor for at least 5 min before measuring blood pressure (12). A standard mercury sphygmomanometer was used to record systolic blood pressure (SBP) and diastolic blood pressure (DBP) at the first and the fifth phase Korotkoff sounds. The average of two sequential blood pressure values, with a minimum of a 2-min rest period between measures, was recorded. Using the average of these two sequential blood pressure values, the mean SBP and DBP were calculated (13). For our research purposes, DBP ≥ 90 mmHg was defined as diastolic hypertension, and SBP ≥ 140 mmHg was defined as systolic hypertension (13).

### Daily Dietary Estimate

A modified 1995 Block Food Frequency Questionnaire (FFQ) was used to evaluate daily dietary intake with 103 food items (14, 15) based on the Second National Health and Nutrition Examination Survey (NHANES) (14, 16, 17). Four indices of DDE of dietary fiber (dietary fiber intake, dietary fiber intake from beans, dietary fiber intake from vegetables/fruit, and dietary fiber intake from grains) were computed based on a database of food composition from the data of the United States Department of Agriculture (USDA) linked to data of food frequency (18).

### Covariates

For research purposes, body mass index (BMI) was calculated as weight (kg) divided by height (meters) squared. Smoking status was divided into “current smoker” and “not current smoker”. Medical history (cholesterol medications ever taken, blood pressure medications ever taken, diuretics ever taken, and birth control pills ever taken) was classified as “Yes” and “No”. In addition, biochemical parameters including blood C-reactive protein (CRP), total cholesterol, triglycerides, low-density lipoprotein cholesterol (LDL-C), high-density lipoprotein cholesterol (HDL-C), glucose, and insulin were detected in the SWAN study.

### Statistical Analysis

First, we performed a smooth curve to estimate the associations of four indices of DDE of dietary fiber (dietary fiber intake, dietary fiber intake from beans, dietary fiber intake from vegetables/fruit, and dietary fiber intake from grains) with blood pressure. Then, multiple linear regression models were used to explore the associations between these four fiber indices and SBP and DBP. Furthermore, multiple logistic regression models were also used to explore the relationships between the four DDE fiber indices and systolic hypertension (SBP ≥ 140 mm Hg) and diastolic hypertension (DBP ≥ 90 mm Hg). In addition, we implemented a stratified analysis to estimate the relationships between the four fiber indices and blood pressure stratified by age, race, BMI, and smoking status. In multivariate analysis, the following five models were used: Crude Model: adjustment for nothing; Model 1: adjustment for age and race; Model 2: adjustment for age, race, DDE fat, DDE carb, and DDE protein; Model 3: adjustment for age, race, DDE fat, carb, protein, smoking status, and BMI; and Model 4: adjustment for age, race, DDE fat, carb, protein, smoking status, BMI, blood CRP, total cholesterol, triglycerides, LDL-C, HDL-C, glucose, and insulin. All of the analyses were performed using EmpowerStats 3.0 (Chinese version). A *P* ≤ 0.05 was considered to be statistically significant.

## Results

### Population Characteristics

A total of 2,519 subjects were included, with an average age of 46. As shown in Table 1, the mean values of SBP and DBP were 114 and 74 mmHg, respectively. The mean values of DDE dietary fiber, DDE fiber from beans, DDE fiber from vegetables/fruit, and DDE fiber from grains in the included participants were 11.52, 1.22, 5.36, and 3.91, respectively.

**Table 1:** Characteristics of participants (*n* = 2519).

**Variables**	**Mean ± SD or %**	**Range or N**
Age (years)	46 ± 2.69	42–53
**Race**		
Black/African American (%)	27.79	700
Chinese/Chinese American (%)	7.90	199
Japanese/Japanese American (%)	8.50	214
Caucasian/White Non-Hispanic (%)	48.91	1232
Hispanic	6.91	174
**Physical parameters**		
Height (cm)	162.40 ± 6.75	140.50–186.20
Weight (kg)	70.20 ± 20.24	37.60–172.10
Waist circumference (cm)	82.5 ± 15.67	59–154.30
Hip circumference (cm)	103.80 ± 14.74	74–173
BMI (kg/m2)	26.47 ± 7.18	14.99–64.83
Smoking status (current smoker), (%)	16.71	421
Average DBP (mmHg)	74.00 ± 10.38	41–144
High DBP (≥90mmHg), (%)	9.83	147
Average SBP (mmHg)	114.00 ±16.88	74–224
High SBP (≥140mmHg), (%)	10.13	253
**Medical history**		
Cholesterol medications ever taken (%)	0.95	24
Blood pressures medications ever taken (%)	11.23	283
Diuretics ever taken (%)	9.09	229
Birth control pills ever taken (%)	72.89	1836
**DDE nutrients**		
DDE fat (g/day)	61.75 ± 30.86	12.04–218.67
DDE carb (g/day)	215.92 ± 98.66	18.75–792.56
DDE protein (g/day)	66.74 ± 25.18	17.49–204.49
DDE dietary fiber (g/day)	11.52 ± 5.79	1.66–61.78
DDE fiber from beans (g/day)	1.22 ± 2.46	0–39.62
DDE fiber from vegetables/fruit (g/day)	5.36 ± 3.55	0.60–26.85
DDE fiber from grains (g/day)	3.91 ± 2.16	0.10–16.61
**Blood biomarkers**		
Total cholesterol (mg/dl)	191 ± 33.75	92–335
HDL-C (mg/dl)	54 ± 14.41	18–166
LDL-C (mg/dl)	114 ± 30.60	25–261
Triglycerides (mg/dl)	89 ± 57.29	31–395
Lipoprotein A-1 (mg/dl)	48 ± 11.42	20–122
Apolipoprotein A-1 (mg/dl)	148 ± 24.95	73–317
Apolipoprotein B (mg/dl)	108 ± 28.41	73–317
CRP (mg/l)	1.50 ± 6.22	0.04–105.60
Glucose (mg/dl)	91 ± 30.45	45–439
Insulin (uIU/ml)	8.50 ± 13.17	2.50–417.20

*DBP, diastolic blood pressure; SBP, systolic blood pressure; DDE, daily dietary estimate; BMI, body mass index. CRP, C-reactive protein; LDL-C, low-density lipoprotein cholesterol; HDL-C, high-density lipoprotein cholesterol*.

**Figure 2:**
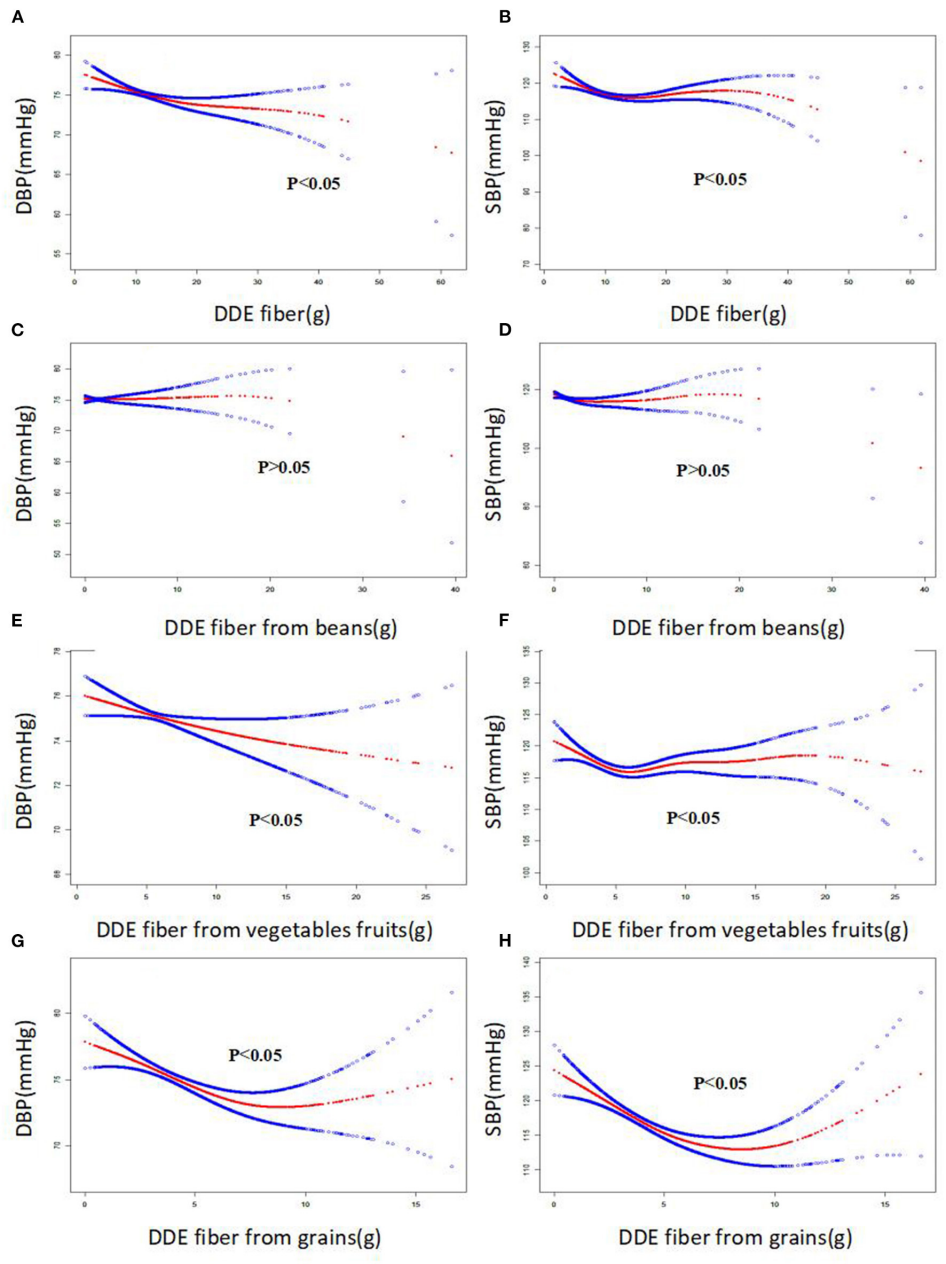
The smooth curve of the associations between dietary fiber intake and systolic blood pressure and diastolic blood pressure (SBP and DBP, respectively).

**Figure 3:**
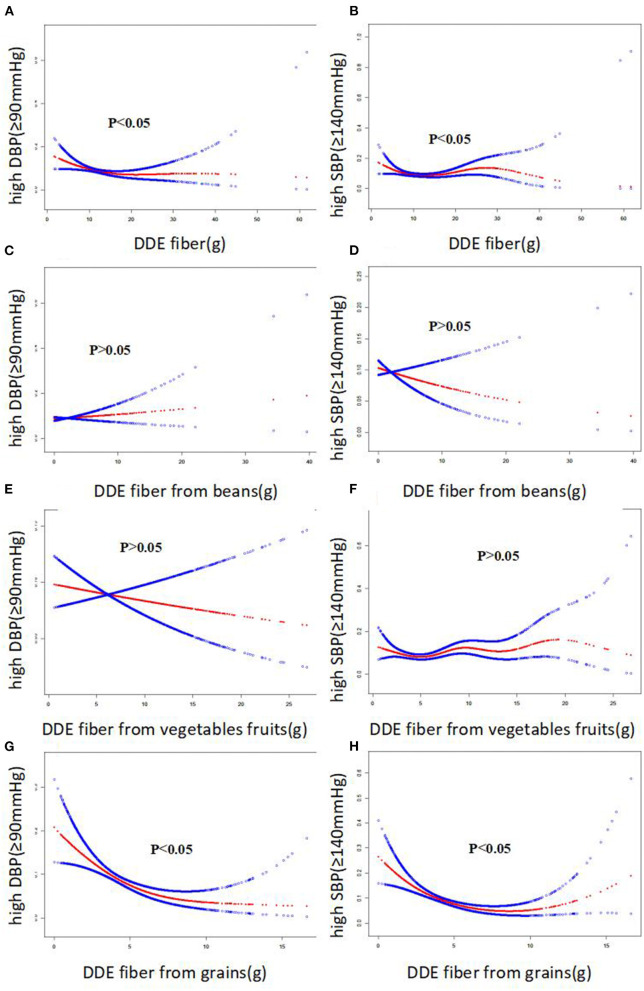
The smooth curve of the associations between dietary fiber intake and risk of systolic and diastolic hypertension, respectively.

Smooth curves were identified between the four DDE fiber indices and blood pressure. The smooth curve suggested approximate negative correlations between three fiber indices (DDE dietary fiber, DDE fiber from vegetables/fruit, and DDE fiber from grains) and blood pressure, including DBP and SBP (Figure 2, all *P* < 0.005). Importantly, there were approximate negative correlations between two fiber indices (DDE dietary fiber and DDE fiber from grains) and the risk of diastolic hypertension and systolic hypertension (Figure 3, all *P* < 0.05).

### Association Between Dietary Fiber Intake and BP

To explore the correlation between the intake of dietary fiber and blood pressure, a multiple linear regression analysis was performed. As shown in Table 2, after adjusting for confounding factors, including age, race, DDE fat, carb, protein, smoking status, BMI, blood CRP, glucose, insulin, LDL-C, HDL-C, triglycerides and total cholesterol, DDE dietary fiber (Sβ = −0.057, 95% CI −0.194 – −0.012, *P* = 0.027), DDE fiber from vegetables/fruit (Sβ = −0.046, 95% CI −0.263 – −0.007, *P* = 0.039), and DDE fiber from grains (Sβ = −0.073, 95% CI −0.600 – −0.099, *P* = 0.006) were still negatively correlated with DBP in Model 4. However, we observed that only DDE fiber from grains was independently and negatively associated with SBP (Sβ = −0.060, 95% CI −0.846 – −0.093, *P* = 0.015) after these same confounding factors were adjusted.

**Table 2:** Multivariate linear regression on association of DDE fiber and blood pressure.

**Variables**	**DBP (mmHg)**				**SBP (mmHg)**			
	**B**	**Sβ**	**B 95% CI**	***P* Value**	**B**	**Sβ**	**B 95% CI**	***P* Value**
**Crude**								
DDE dietary fiber	−0.085	−0.047	−0.155, −−0.015	0.018	−0.134	−0.046	−0.247, −−0.020	0.021
DDE fiber from beans	−0.025	−0.006	−0.190, 0.140	0.766	−0.270	−0.039	−0.537, −−0.003	0.048
DDE fiber from vegetables/fruit	−0.117	−0.040	−0.231, −−0.003	0.045	−0.060	−0.013	−0.246, 0.125	0.523
DDE fiber from grains	−0.239	−0.050	−0.427, −−0.052	0.012	−0.418	−0.054	−0.723, −−0.113	0.007
**Model 1**								
DDE dietary fiber	−0.087	−0.048	−0.156, −−0.017	0.015	−0.136	−0.047	−0.246, −−0.027	0.015
DDE fiber from beans	0.002	0.000	−0.163, 0.166	0.985	−0.103	−0.015	−0.363, 0.156	0.435
DDE fiber from vegetables/fruit	−0.140	−0.048	−0.254, −−0.027	0.016	−0.161	−0.034	−0.340, 0.018	0.077
DDE fiber from grains	−0.227	−0.047	−0.414, −−0.041	0.017	−0.380	−0.049	−0.673, −−0.086	0.011
**Model 2**								
DDE dietary fiber	−0.156	−0.087	−0.248, −−0.065	0.001	−0.219	−0.075	−0.363, −−0.075	0.003
DDE fiber from beans	−0.001	0.000	−0.175, 0.172	0.987	−0.079	−0.012	−0.352, 0.194	0.571
DDE fiber from vegetables/fruit	−0.175	−0.060	−0.305, −−0.044	0.009	−0.148	−0.031	−0.353, 0.057	0.157
DDE fiber from grains	−0.491	−0.102	−0.744, −−0.238	<0.001	−0.882	−0.113	−1.279, −−0.485	<0.001
**Model 3**								
DDE dietary fiber	−0.109	−0.061	−0.200, −−0.017	0.020	−0.062	−0.021	−0.200, 0.076	0.376
DDE fiber from beans	0.055	0.013	−0.116, 0.226	0.529	0.092	0.013	−0.166, 0.350	0.486
DDE fiber from vegetables/fruit	−0.137	−0.047	−0.266, −−0.008	0.037	−0.023	−0.005	−0.218, 0.171	0.813
DDE fiber from grains	−0.382	−0.080	−0.633, −−0.130	0.003	−0.528	−0.068	−0.907, −−0.150	0.006
**Model 4**								
DDE dietary fiber	−0.103	−0.057	−0.194, −−0.012	0.027	−0.062	−0.021	−0.199, 0.075	0.372
DDE fiber from beans	0.056	0.013	−0.115, 0.226	0.523	0.083	0.012	−0.174, 0.339	0.527
DDE fiber from vegetables/fruit	−0.135	−0.046	−0.263, −−0.007	0.039	−0.035	−0.007	−0.228, 0.158	0.720
DDE fiber from grains	−0.349	−0.073	−0.600, −−0.099	0.006	−0.469	−−0.060	−−0.846, −−0.093	0.015

*Crude: Adjusted for nothing. Model 1: Adjusted for age and race. Model 2: Adjusted for age, race, DDE fat, DDE carb and DDE protein. Model 3: Adjusted for age, race, DDE fat, carb, protein, smoking status, BMI. Model 4: Adjusted for age, race, DDE fat, carb, protein, smoking status, BMI, blood CRP, total cholesterol, triglycerides, LDL-C, HDL-C, glucose and insulin. DBP, diastolic blood pressure; SBP, systolic blood pressure; DDE, daily dietary estimate; BMI, body mass index. CRP, C-reactive protein; LDL-C, low-density lipoprotein cholesterol; HDL-C, high-density lipoprotein cholesterol*.

### Association Between the Intake of Dietary Fiber and Hypertension Risk

Multiple logistic regression models were used to further prove the correlation between dietary fiber intake and hypertension risk. As shown in Table 3, our results only suggested that higher DDE fiber from grains was independently associated with a reduced risk of diastolic hypertension (OR = 0.848, 95% CI 0.770–0.934, *P* = 0.001, Model 4) after adjustments were made for confounding factors, including age, race, DDE fat, carb, protein, smoking status, BMI, blood CRP, glucose, insulin, LDL-C, HDL-C, triglycerides, and total cholesterol. Similarly, our results also showed that higher DDE fiber from grains was associated with a reduced risk of systolic hypertension (OR = 0.906, 95% CI 0.826–0.993, *P* = 0.034, Model 4) after adjusting for these same confounding factors.

**Table 3:** Multivariate logistic regression on association of DDE fiber and risk of hypertension.

**Variables**	**High DBP (≥90mmHg)**			**High SBP (≥140mmHg)**		
	**OR**	**95% CI**	***P* Value**	**0R**	**95% CI**	***P* Value**
**Crude**						
DDE dietary fiber	0.981	0.956, 1.006	0.132	0.999	0.976, 1.022	0.909
DDE fiber from beans	1.018	0.967, 1.071	0.499	0.963	0.905, 1.026	0.246
DDE fiber from vegetables/fruit	0.980	0.941, 1.020	0.328	1.029	0.994, 1.066	0.108
DDE fiber from grains	0.895	0.834, 0.961	0.002	0.950	0.891, 1.013	0.120
**Model 1**						
DDE dietary fiber	0.981	0.957, 1.006	0.140	1.001	0.978, 1.025	0.948
DDE fiber from beans	1.031	0.981, 1.084	0.227	0.998	0.939, 1.060	0.942
DDE fiber from vegetables/fruit	0.973	0.934, 1.013	0.186	1.016	0.980, 1.053	0.398
DDE fiber from grains	0.901	0.840, 0.967	0.004	0.956	0.906, 0.996	0.039
**Model 2**						
DDE dietary fiber	0.971	0.938, 1.005	0.092	0.985	0.953, 1.017	0.341
DDE fiber from beans	1.036	0.983, 1.092	0.185	0.992	0.929, 1,058	0.799
DDE fiber from vegetables/fruit	0.972	0.928, 1.019	0.239	1.010	0.968, 1.053	0.652
DDE fiber from grains	0.823	0.748, 0.906	<0.001	0.871	0.796, 0.954	0.003
**Model 3**						
DDE dietary fiber	0.983	0.950, 1.017	0.333	1.003	0.993, 1.037	0.837
DDE fiber from beans	1.051	0.998, 1.108	0.061	1.018	0.955, 1.085	0.585
DDE fiber from vegetables/fruit	0.982	0.937, 1.029	0.436	1.024	0.981, 1.069	0.280
DDE fiber from grains	0.845	0.767, 0.930	0.001	0.906	0.827, 0.992	0.032
**Model 4**						
DDE dietary fiber	0.984	0.951, 1.018	0.355	1.003	0.970, 1.037	0.859
DDE fiber from beans	1.053	0.999, 1.110	0.057	1.019	0.954, 1.088	0.580
DDE fiber from vegetables/fruit	0.982	0.937, 1.029	0.436	1.023	0.979, 1.068	0.308
DDE fiber from grains	0.848	0.770, 0.934	0.001	0.906	0.826, 0.993	0.034

*Crude: Adjusted for nothing. Model 1: Adjusted for age and race. Model 2: Adjusted for age, race, DDE fat, DDE carb and DDE protein. Model 3: Adjusted for age, race, DDE fat, carb, protein, smoking status, BMI. Model 4: Adjusted for age, race, DDE fat, carb, protein, smoking status, BMI, blood CRP, total cholesterol, triglycerides, LDL-C, HDL-C, glucose and insulin. DBP, diastolic blood pressure; SBP, systolic blood pressure; DDE, daily dietary estimate; BMI, body mass index. CRP, C-reactive protein; LDL-C, low-density lipoprotein cholesterol; HDL-C, high-density lipoprotein cholesterol*.

### Stratified Analysis of the Association Between DDE Fiber From Grains and Blood Pressure by Age, Race, BMI, and Smoking Status

Multiple linear regression models were used to further explore whether the associations between DDE fiber from grains and blood pressure were affected by sex, race, BMI, and smoking status. As shown in Table 4, we observed no significant effect modifiers between DDE fiber from grains and DBP after stratifying the analysis by age (interaction *P* = 0.724), race (interaction *P* = 0.125), BMI (interaction *P* = 0.815), and smoking status (interaction *P* = 0.347). We also observed no significant effect modifiers between DDE fiber from grains and SBP after stratification of the analysis by age (interaction *P* = 0.629), race (interaction *P* = 0.114), BMI (interaction *P* = 0.829), and smoking status (interaction *P* = 0.664).

**Table 4:** Multiple linear regression analysis for relationship between DDE fiber from grains and blood pressure stratified by age, race, BMI and current smoker prespectively.

**Subgroup**	**DBP (mmHg)**			**SBP (mmHg)**		
	**B, 95%CI**	***P***	**Interaction P**	**B, 95%CI**	***P***	**Interaction P**
**Age**						
<46	−0.45 (−0.81, −0.10)	0.012	0.724	−0.58 (−1.12, −0.03)	0.037	0.629
≥46	−0.36 (−0.71, −0.01)	0.041		−0.76 (−1.30, −0.23)	0.005	
**Race**						
White	−0.04 (−0.39, 0.31)	0.817	0.125	0.09 (−0.44, 0.62)	0.736	0.14
No–white	−0.43 (−0.79, −0.07)	0.018		−0.85 (−1.39, −0.31)	0.002	
**BMI**						
BMI <25 (normal)	−0.41 (−0.82, −0.01)	0.045	0.85	−0.77 (−1.40, −0.14)	0.016	0.829
BMI≥25 (overweight)	−0.35(−0.67, −0.03)	0.030		−0.68 (−1.18, −0.19)	0.006	
**Smoking status (current smoker)**						
No	−0.32 (−0.59, −0.06)	0.017	0.37	−0.53 (−0.94, −0.13)	0.009	0.64
Yes	−0.56 (−1.03, −0.08)	0.022		−0.70 (−1.42, −0.01)	0.048	

*Adjusted for age, race, DDE fat, carb, protein, smoking status, BMI, blood CRP, total cholesterol, triglycerides, LDL-C, HDL-C, glucose and insulin. DBP, diastolic blood pressure; SBP, systolic blood pressure; DDE, daily dietary estimate; BMI, body mass index. CRP, C-reactive protein; LDL-C, low-density lipoprotein cholesterol; HDL-C, high-density lipoprotein cholesterol*.

Multiple logistic regression models were also performed to further investigate whether the associations between DDE fiber from grains and the risk of hypertension were affected by sex, race, BMI, and smoking status (Table 5). We observed no significant effect modifiers between DDE fiber from grains and the risk of diastolic hypertension after the stratified analysis by age (interaction *P* = 0.898), race (interaction *P* = 0.225), BMI (interaction *P* = 0.716), or smoking status (interaction *P* = 0.450). We also observed no significant effect modifiers between DDE fiber from grains and the risk of systolic hypertension after the stratified analysis by race (interaction *P* = 0.265), BMI (interaction *P* = 0.117), or smoking status (interaction *P* = 0.221). Importantly, age (<46 and ≥46 years) was an effect modifier of the association between DDE fiber from grains and the risk of systolic hypertension (interaction *P* = 0.009).

**Table 5:** Multiple logistic regression analysis for relationship between DDE fiber from grains and risk of hypertension stratified by age, race, BMI and current smoker prespectively.

**Subgroup**	**High DBP (≥90 mmHg)**			**HighSBP (≥140 mmHg)**		
	**OR, 95%CI**	***P***	**Interaction P**	**OR, 95%CI**	***P***	**Interaction P**
**Age**						
<46	0.82 (0.71, 0.96)	0.010	0.898	0.73 (0.62, 0.86)	<0.001	0.009
≥46	0.84 (0.74, 0.95)	0.005		0.95 (0.85, 1.06)	0.334	
**Race**						
White	0.96 (0.80, 1.15)	0.652	0.225	1.00 (0.84, 1.19)	0.975	0.265
Others	0.84 (0.75, 0.94)	0.003		0.89 (0.80, 0.99)	0.035	
**BMI**						
BMI <25 normal	0.81 (0.65, 1.00)	0.050	0.716	0.74 (0.58, 0.93)	0.011	0.117
BMI ≥25 overweight	0.84 (0.76, 0.93)	0.001		0.90 (0.82, 0.99)	0.023	
**Smoking status (current smoker)**						
No	0.86 (0.77, 0.95)	0.005	0.450	0.92 (0.83, 1.01)	0.093	0.221
Yes	0.79 (0.65, 0.96)	0.020		0.81 (0.69, 0.96)	0.017	

*Adjusted for age, race, DDE fat, carb, protein, smoking status, BMI, blood CRP, total cholesterol, triglycerides, LDL-C, HDL-C, glucose and insulin. DBP, diastolic blood pressure; SBP, systolic blood pressure; DDE, daily dietary estimate; BMI, body mass index. CRP, C-reactive protein; LDL-C, low-density lipoprotein cholesterol; HDL-C, high-density lipoprotein cholesterol*.

## Discussion

Our study included baseline data from SWAN including 2,519 subjects to investigate the correlation between dietary fiber intake and hypertension risk. Our results uncovered that DDE fibers from grains were negatively associated with the risk of hypertension after adjusting for confounders. This is the first study to show that increased intake of DDE fiber from grains may contribute to a reduced risk of hypertension in midlife women.

Dietary fiber intake has great benefits for preventing many chronic diseases, such as cardiovascular diseases and malignant tumors. Studies on the beneficial effect of dietary fiber on blood pressure began to be investigated in experimental animals. For example, previous evidence has suggested that increased intake of certain fibers has beneficial effects on improved blood pressure in rats (19). Obata et al. observed that one type of fiber, the husk of Psyllium seeds, can lower blood pressure in hypertensive rats who were fed a high-salt diet (19). A few years later, Li et al. performed a study on model rats who were fed corn starch, white rice, and rye for 16 weeks. They found that the rye diet lowered SBP significantly after 12 weeks of treatment. This diet also lowered serum levels of triglycerides, LDL cholesterol, and total cholesterol in the animals. They concluded that the intake of dietary fiber could contribute beneficial effects to blood lipids and blood pressure (20). Galisteo et al. also found a similar result in obese rats fed a diet containing 3.5% Plantago ovata for 25 weeks (21). They observed that consumption of a fiber-enriched diet could prevent hypertension, endothelial dysfunction, and obesity in rats. Recently, one study also reported that the short-term intake of cocoa fiber products could lower blood pressure in rats (22).

For the clinical studies, for example, Saltzman and his team investigated the role of oat fiber in hypertension (23). They observed that an oat-rich diet did contribute to a reduction in SBP without changing the levels of DBP (23). In addition, the diet also decreased the levels of blood lipids. Keenan et al. observed that oat cereal supplements could lower the need for improving arterial blood pressure and antihypertensive medication in hypertensive patients (24), and also found beneficial effects of oat fiber in hypertensive patients with grade 1 hypertension. These patients were given soluble oat fiber for 3 months, and this fiber led to a significant decrease in blood pressure (25). Consistent with these previous findings and mechanisms, we found that increased dietary fiber intake, especially DDE fiber from grains, is related to a lower risk of systolic hypertension and diastolic hypertension in midlife women. However, we did not find that increasing the intake of dietary fiber from beans and vegetables/fruit was associated with reduced hypertension risk. Elevated dietary fiber intake from grains contributed to a decreased risk of hypertension. This is a very interesting discovery, and more future studies may be necessary to explain the difference.

However, the potential antihypertensive mechanisms of dietary fiber intake have not been elucidated until now. Insulin resistance has been found to be associated with the development of hypertension, and dietary fiber intake might improve blood pressure by modulating insulin metabolism (26, 27). Reduced levels of serum cholesterol are associated with improved endothelial function, which mediates vasodilation and reduces blood pressure (28–30). The weight loss caused by dietary fiber has also been considered the underlying mechanism for the lowering of high blood pressure (31–33). However, these known mechanisms are not enough to explain the protective effect of dietary fiber intake on improving blood pressure. Therefore, more studies are necessary to fully elucidate new mechanisms.

Our study has several notable strengths. First, our study results were from the SWAN study, a multicenter and population-based study of the natural history of late midlife women. The SWAN study has high-quality data, including demographic characteristics, lifestyle, self-reported health, health examination, and medical history, with various sampling frames and recruitment strategies. Second, we comprehensively analyzed the relationship between the main sources of dietary fiber (beans, fruits/vegetables, and grains) and blood pressure. In this cross-sectional study, we are the first to find that increased intake of DDE fiber from grains, rather than the intake of DDE fiber from beans and fruits/vegetables, contributed to a reduced risk of hypertension in midlife women from the United States. Third, enough confounding factors, including demographic characteristics and lifestyle, and biochemical indices, were adjusted by multivariable analysis, which ensures the reliability of our results.

Of course, this study also has several limitations. On the one hand, our data did not distinguish between essential hypertension and secondary hypertension. The diagnosis of hypertension was determined by temporary blood pressure measurement at baseline in the SWAN study. The positive effect of dietary fiber on secondary hypertension caused by other diseases may be insignificant. On the other hand, the results of our study are only applicable to midlife women. The effect of dietary fiber on lowering blood pressure in men and different age groups (minors and the elderly) should be further confirmed in the future. In addition, although 3,302 subjects were included in the SWAN study, only 2,519 subjects were eventually included in our study because approximately 783 individuals were excluded due to missing data.

## Conclusion

Our results further suggested that fiber intake, especially the DDE fiber from grains, is associated with the risk of hypertension after controlling for potential confounders.

## Data Availability Statement

The original contributions presented in the study are included in the article/supplementary files, further inquiries can be directed to the corresponding author/s.

## Ethics Statement

The studies involving human participants were reviewed and approved by The institutional review boards at all sites have approved the study protocol and all included individual at each site gave informed consent. The patients/participants provided their written informed consent to participate in this study.

## Author Contributions

All authors contributed to the article and approved the submitted version.

## Funding

This study was supported by grants from the Natural Science Foundation of Jiangxi Province Youth Fund Project (20192BAB215012), the Science and Technology Research Youth Project of Education Department of Jiangxi Province (GJJ180124), the Natural Science Foundation of China (82060122), and the Key Project of Jiangxi Provincial Department of Education (GJJ180014).

## Conflict of Interest

The authors declare that the research was conducted in the absence of any commercial or financial relationships that could be construed as a potential conflict of interest.

## Publisher's Note

All claims expressed in this article are solely those of the authors and do not necessarily represent those of their affiliated organizations, or those of the publisher, the editors and the reviewers. Any product that may be evaluated in this article, or claim that may be made by its manufacturer, is not guaranteed or endorsed by the publisher.
